# Evaluation of a commercial Monte Carlo dose calculation algorithm for electron treatment planning

**DOI:** 10.1002/acm2.12622

**Published:** 2019-05-23

**Authors:** Jessie Y. Huang, David Dunkerley, Jennifer B. Smilowitz

**Affiliations:** ^1^ Department of Human Oncology University of Wisconsin‐Madison Madison WI USA

**Keywords:** electron treatment planning, heterogeneous dose calculation, Monte Carlo, MPPG 5.a validation

## Abstract

The RayStation treatment planning system implements a Monte Carlo (MC) algorithm for electron dose calculations. For a TrueBeam accelerator, beam modeling was performed for four electron energies (6, 9, 12, and 15 MeV), and the dose calculation accuracy was tested for a range of geometries. The suite of validation tests included those tests recommended by AAPM's Medical Physics Practice Guideline 5.a, but extended beyond these tests in order to validate the MC algorithm in more challenging geometries. For MPPG 5.a testing, calculation accuracy was evaluated for square cutouts of various sizes, two custom cutout shapes, oblique incidence, and heterogenous media (cork). In general, agreement between ion chamber measurements and RayStation dose calculations was excellent and well within suggested tolerance limits. However, this testing did reveal calculation errors for the output of small cutouts. Of the 312 output factors evaluated for square cutouts, 20 (6.4%) were outside of 3% and 5 (1.6%) were outside of 5%, with these larger errors generally being for the smallest cutout sizes within a given applicator. Adjustment of beam modeling parameters did not fix these calculation errors, nor does the planning software allow the user to input correction factors as a function of field size. Additional validation tests included several complex phantom geometries (triangular nose phantom, lung phantom, curved breast phantom, and cortical bone phantom), designed to test the ability of the algorithm to handle high density heterogeneities and irregular surface contours. In comparison to measurements with radiochromic film, RayStation showed good agreement, with an average of 89.3% pixels passing for gamma analysis (3%/3mm) across four phantom geometries. The MC algorithm was able to accurately handle the presence of irregular surface contours (curved cylindrical phantom and a triangular nose phantom), as well as heterogeneities (cork and cortical bone).

## 1 INTRODUCTION

In comparison to pencil beam electron dose calculation algorithms, the use of Monte Carlo algorithms for electron treatment planning has been shown to improve dose calculation accuacy.^1^, ^2^ Significant differences in the dose distributions calculated using a pencil beam algorithm vs a Monte Carlo algorithm were found for electron treatment plans with challenging geometries, i.e., regions near air cavities and bones, extended source to surface distance(SSDs) oblique incidence, and small irregular fields.^3^, ^4^ In comparison to photon treatment planning, electron Monte Carlo (MC) dose calculation algorithms are more computationally efficient, requiring fewer histories to achieve a given statistical uncertainty. The relative speed and improved accuracy of electron MC algorithms has allowed their introduction into commercial treatment planning systems (TPS) in recent years.

The RayStation TPS implements a MC dose calculation algorithm for electron treatment planning that uses the VMC++ Monte Carlo code for in‐patient energy transport and scoring.^5^ VMC++ is a voxel‐based Monte Carlo code that has been incorporated into several commercial planning systems, including Oncentra MasterPlan® and CMS XiO®. The RayStation implementation of this MC algorithm models several beamline components, including the jaws, MLCs, electron applicator scraper layers, and the electron cutout, in the generation of phase space data which is then fed into the VMC++ code in order to perform dose calculations in the patient geometry.

Several guidance documents exist to aid medical physicists in developing tests to validate and commission an electron MC dose calculation algorithm for treatment planning purposes. The AAPM Medical Physics Practice Guideline 5.a (MPPG 5.a) recommends validation tests for electron beams, including comparison of calculated vs. measured dose distributions for standard cutouts, custom cutouts at standard and extended SSDS, oblique incidence, and inhomogeneous phantom geometries.^6^ Though the suite of tests recommended by MPPG 5.a represents the minimum testing that should be performed to commission an electron dose calculation algorithm, the tests are typically performed using simple geometries (e.g., water tank and a cork slab phantom) that do not fully explore the accuracy of more advanced Monte Carlo algorithms. AAPM Task Group Report No. 105 addresses issues specifically associated with Monte Carlo–based treatment planning algorithms; this task group report states that beam model validation should include measurements in heterogeneous phantom geometries, similar to those reported in the Electron Collaborative Working Group report.^1^, ^7^, ^8^ The more complex validation phantom geometries used by the Electron Collaborative Working Group include irregular surface contours (e.g., nose‐shaped phantoms, stepped‐surface phantoms) as well as internal 3D heterogeneities (e.g., bone and air cavities). Cygler et al. (2003) evaluated the VMC++ code using phantom geometries of varying degrees of complexity, including 1D (slab), 2D (rib), and 3D (small cylindrical) heterogeneities, as well as a complex phantom geometry designed to mimic the trachea and the spine.^9^

Though studies have been done to quantify the accuracy of electron Monte Carlo algorithms in complex heterogeneous phantom geometries, very little has been published on RayStation’s implementation of electron MC. Archibald‐Herren and Liu *et al.* evaluated the accuracy of RayStation’s electron MC algorithm for obliquely incident beams on a water phantom.^10^ However, to the authors’ knowledge, there are no published studies that have performed a comprehensive analysis of the calculation accuracy of RayStation’s electron MC algorithm that includes complex heterogeneous phantom geometries. Thus, the purpose of this study is to describe the full suite of testing undertaken to evaluate the accuracy of RayStation’s electron MC algorithm in order to commission the system for clinical use. This testing extends beyond that recommended in MPPG 5.a, with complex phantom geometries designed specifically to test the increased accuracy of a MC algorithm in the presence of heterogeneities and irregular surface contours. Additionally, other issues relevant for the clinical implementation of the algorithm will be discussed, including the impact of statistical noise in the dose distribution for prescribing and calculating monitor units as well as how to handle discrepancies between Monte Carlo‐calculated monitor units and secondary MU calculation software.

## 2 MATERIALS AND METHODS

### 2.1 RayStation beam modeling

RayStation’s electron dose calculation algorithm utilizes phase space data in order to perform Monte Carlo dose calculations. The electron beam within the linear accelerator is modeled using a “source phase space” at the level of the secondary scattering foils. The source phase space electron particles are then propagated through the beamline components in the linear accelerator (jaws, MLCs, electron applicator, and electron cutout) in order to generate an “exit phase space” which is then used for Monte Carlo transport through the patient geometry; the exit phase space is defined at the level of the patient‐specific cutout in the applicator. Therefore, electron beam modeling in RayStation requires the user to enter machine‐specific information in order to model the accelerator beamline for exit phase space generation, including the thickness and position of the MLCs and jaws, information about the electron applicator scraper layers, energy and applicator‐specific jaw settings, primary and secondary scattering foil locations, and cutout thickness. Modeling of the source phase space requires the measurement of in‐air data (without an applicator in place), including in‐air relative output factors and in‐air profiles for various field sizes at 70 and 90 cm SSD. Additional required measurement data for beam modeling includes in‐water depth dose curves measured with and without electron applicators in place, in‐water profiles at two depths for each energy and applicator combination, and absolute calibration data (dose per MU) for each energy and electron applicator combination. In‐water measurements were used to optimize electron spectrum parameters in the beam model, as well as to fine‐tune source phase space parameters.

All beam modeling data were acquired for a TrueBeam linear accelerator for 6, 9, 12, and 15 MeV electron beams using a 3D scanning water tank (Blue Phantom^2^, IBA Dosimetry, Schwarzenbruck, Germany). Profiles, depth dose curves, and in‐air output factors were measured using an electron field diode detector (IBA Dosimetry), while the absolute dose measurements were performed using a 0.04cc ion chamber (IBA Dosimetry).

### 2.2 MPPG 5.a validation

Once beam modeling for all electron energies was completed, the calculation accuracy of the algorithm and beam models was tested according to the recommendations of MPPG 5.a. For MPPG 5.a testing, all profile and depth dose data were acquired using a 3D scanning water tank and a 0.04 cc ion chamber, and all RayStation dose calculations were performed using a 2 mm dose grid and 500,000 histories per cm^2 ^(resulting in a relative uncertainty of <1.0%). Point dose measurements were evaluated using dose difference criteria between calculated and measured dose, while profile data were compared using 1D gamma analysis (3% global dose difference and 3mm distance‐to‐agreement criteria).

Medical Physics Practice Guideline 5.a recommends that output factors for all electron applicators with standard square cutout sizes for each energy be calculated in order to confirm the correct behavior of output as a function of field size and energy. To perform this comparison, output factors were measured using a 0.04 cc ion chamber and compared to RayStation‐calculated output factors for a range of square cutout sizes in each electron applicator for 100, 105, and 110 cm SSD.

In addition to square cutouts, MPPG 5.a also recommends testing two custom cutout shapes at standard and extended SSDs in order to verify the accuracy of the calculated isodose distribution as well as the system’s ability to handle changes in SSD (MPPG 5.a test 8.1). For this test, two clinically relevant cutout shapes were chosen—a small circular cutout approximately 3 cm in diameter and a larger cutout approximately 6 cm × 20 cm in dimension. Point dose measurements were performed near d_max_ for each electron energy. Additionally, depth dose curves and inline and crossline profile data were measured at two depths per electron energy (d_max_ and R_50_) for both 100 and 105 cm SSD. The measurement geometry was reproduced to calculate dose in RayStation for these two custom cutouts.

To evaluate the accuracy of the dose calculation in the presence of beam obliquity (MPPG5.a test 8.2), measured profile data were compared to calculated dose distributions for beams of oblique incidence (20° gantry rotation, standard 10 cm × 10 cm cutout, 105 cm SSD). Depth dose curves, as well as inline and crossline profiles at d_max_ and R_50 _were measured and compared to RayStation calculations.

Lastly, the accuracy in the presence of heterogeneous materials was evaluated using a cork slab phantom geometry with various thicknesses of cork for different electron energies (MPPG 5.a test 8.3). Measurements were acquired downstream of the cork heterogeneity using a parallel plate ion chamber for a 10 cm × 10 cm reference cutout and 100 cm SSD. Point dose measurements were evaluated at d_max_, and the distance‐to‐agreement between measured and calculated R_50_ was also evaluated.

### 2.3 Complex phantom validation

Four complex phantom geometries were used to test the electron Monte Carlo algorithm in RayStation beyond the testing current recommended by MPPG 5.a. These four complex phantom geometries were designed to mimic clinical cases and to be a more challenging test of the algorithm’s ability to handle heterogeneities and irregular surface contours. All four phantoms were made in‐house.

**Figure 1:**
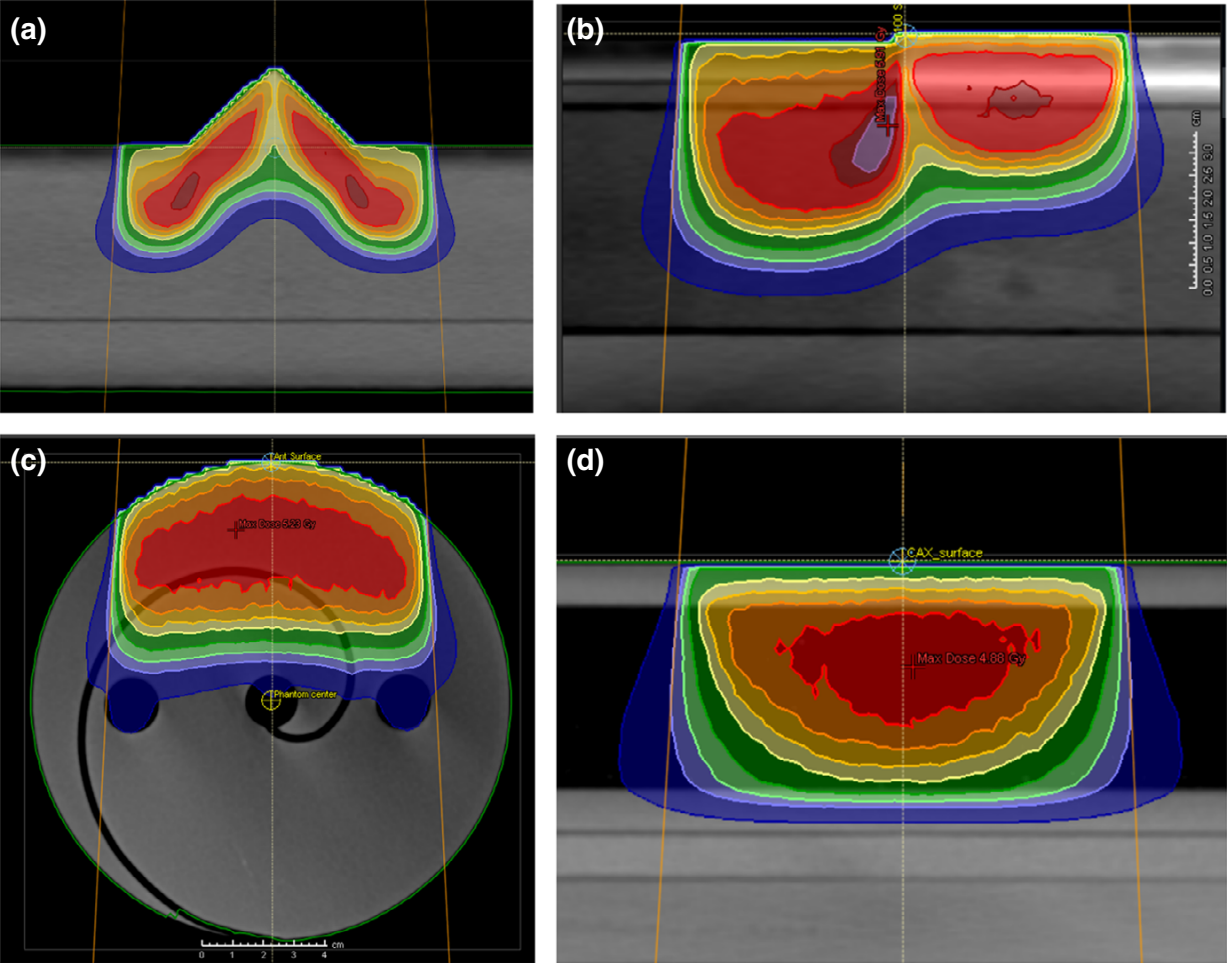
CT images of the four complex validation phantoms with RayStation‐calculated dose for the (a) nose phantom, (b) bone phantom, (c) breast phantom, and (d) lung phantom

- *Nose phantom*: This phantom is composed entirely of solid water with a triangular piece to mimic the geometry of a nose [Fig. 1(a)].
- *Bone phantom*: This rectangular phantom contains a 1 cm slab of cortical bone material (Gammex, Middleton, WI, USA), and the remainder of the phantom is composed of solid water. The central axis of the electron beam is placed such that half of the beam traverses the bone slab, while half of the beam traverses only solid water [Fig. 1(b)].
- *Breast phantom*: This is a cylindrical solid water phantom with a diameter of 15.6 cm. The phantom has halves that are used to sandwich radiochromic film in an edge‐on orientation [Fig. 1(c)]. There is no heterogeneous material in this phantom aside from the air pockets, but it was chosen to somewhat mimic the curvature of a breast for an enface electron beam treatment.
- *Lung phantom*: Similar to the geometry used for MPPG 5.a test 8.3, this rectangular slab phantom is composed of solid water and cork. From the proximal surface, there is 1 cm of solid water, followed by 4 cm of cork, and then 6 cm of solid water [Fig. 1(d)]. Radiochromic film is sandwiched in this phantom in the edge‐on orientation in order to evaluate the algorithm’s ability to predict penumbra broadening within the cork region.

For each of the phantoms, measurements were performed for each electron beam energy (6, 9, 12, and 15 MeV) using Gafchromic EBT3 radiochromic film (Ashland, Inc, Bridgewater, NJ, USA) oriented edge‐on with respect to the beam at 100 cm SSD. 500 MU were delivered per irradiation. A 10 cm x 10 cm standard cutout was used for all measurements. Analysis of the film was performed in the Film QA Pro software (Ashland, Inc., Bridgewater, NJ, USA). A calibration curve was created using known doses ranging from 50 to 800 cGy for 12 MeV. For the other electron energies, the calibration curve was linearly scaled in the film analysis software using experimental films delivered to known doses for that particular energy (600 cGy). Dose calculations were performed in RayStation to match the experimental conditions, with 500,000 histories per cm^2^ and a uniform 2 mm dose grid. The calculated dose exported from the RayStation planning system was then manually registered to the film‐measured dose using isodose lines (IDLs) as a guide to maximize the agreement. Gamma analysis was used to compare the film‐measured dose against RayStation‐calculated dose using the following criteria: 3% global dose difference, 3 mm distance‐to‐agreement, and a 10% dose threshold.

### 2.4 Clinical implementation considerations

Clinical cases, which were originally planned in the Pinnacle TPS (v9.8, Philips Healthcare, Fitchburg, WI, USA) with a pencil beam algorithm, were used to investigate the statistical uncertainty of RayStation Monte Carlo‐calculated dose distributions as well as the impact of this statistical uncertainty on prescription methods. Additionally, secondary MU calculations for these clinical cases were performed using the Mobius3D secondary calculation software (Mobius Medical Systems, LP, Houston, TX, USA). The Electron QuickCalc feature in Mobius3D performs a MU calculation using a pencil beam algorithm and a homogenous water phantom geometry. Given the more accurate handling of patient heterogeneities, irregular surface contour, and oblique incidence in the Monte Carlo algorithm and the difference in sophistication between the RayStation algorithm and the Mobius3D algorithm, it is expected that greater differences in MU between RayStation and Mobius3D will arise than previously encountered between Pinnacle and Mobius3D. Clinical recommendations for the number of histories to use for dose calculation, the acceptable level of statistical uncertainty in the resulting dose distribution, prescription methods, and secondary MU calculations were made based on this investigation.

## 3 RESULTS

### 3.1 MPPG 5.a validation

**Figure 2:**
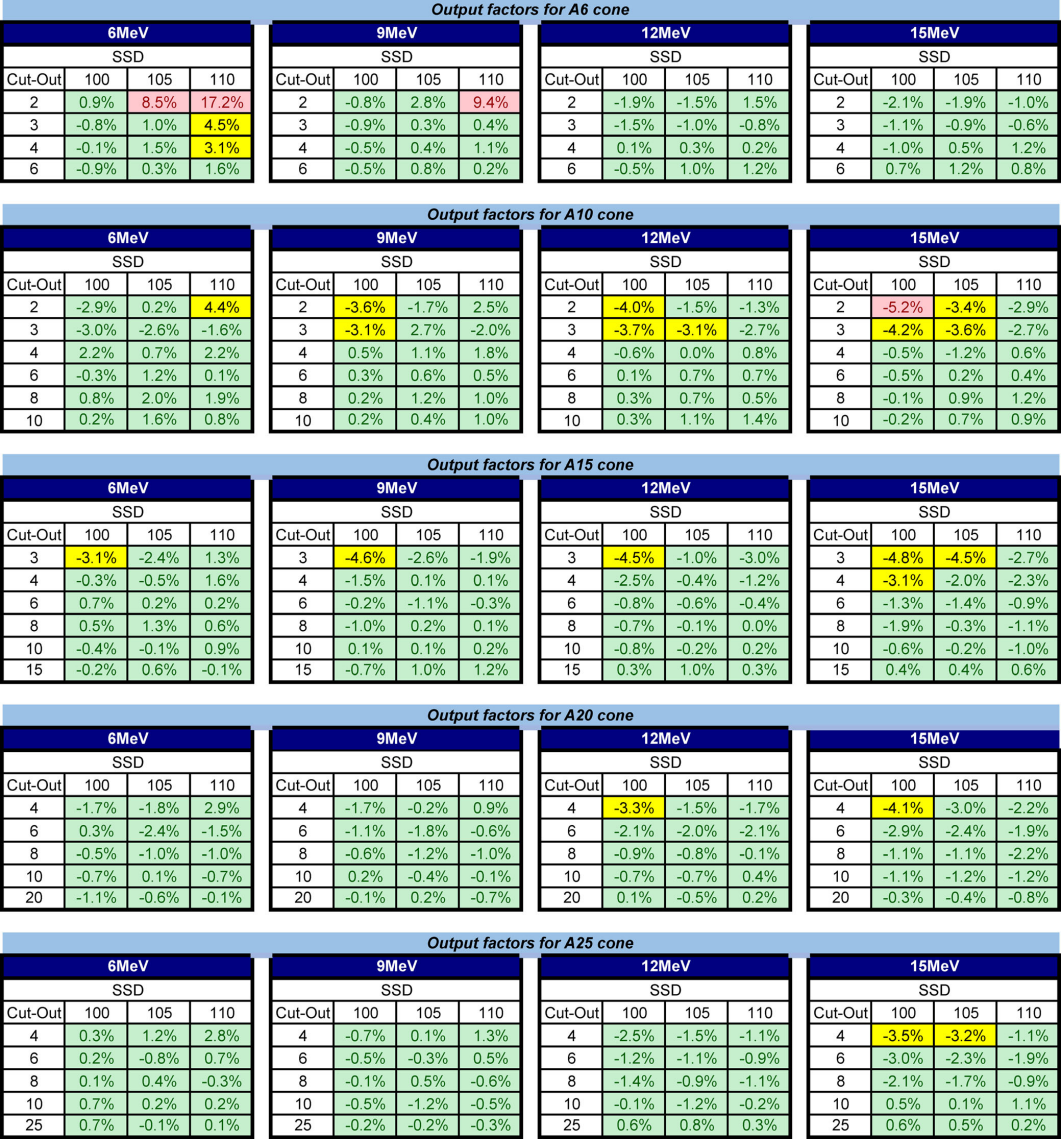
Percent error between RayStation‐calculated output factors and measured output factors for various square cutout sizes, applicator sizes, electron energies, and source to surface distances. Errors > 3% but < 5% are highlighted in yellow, and those exceeding 5% are highlighted in red.

Table 1 shows the results of MPPG 5.a validation for the RayStation electron beam models created for our TrueBeam accelerator, including tests for custom cutouts (one small and one large), oblique incidence, and heterogeneous media (cork slab phantom). In general, the agreement between RayStation dose calculations and measurements was excellent with the exception of the absolute dose for small cutouts. In particular, for MPPG 5.a Test 8.1 for a small custom cutout (3 cm diameter circle), the absolute dose disagreed by > 5% for the 6 MeV beam at 105 cm SSD. Additionally, our comparison of output factors for various square cutout sizes also highlighted issues with small cutouts, especially at extended SSDs with the 6 MeV electron beam. Figure 2 shows the difference between measured output factors and RayStation‐calculated output factors for 100, 105, and 110 cm SSD. Of the 312 output factors evaluated, 20 (6.4%) were outside of 3% and 5 (1.6%) were outside of 5%, with these larger errors generally being for the smallest cutout sizes within a given applicator.

**Table 1:** Summary of MPPG 5.a validation results comparing measurements against RayStation dose calculations for all electron energies (6, 9, 12, and 15 MeV).

MPPG 5.a test	Description	Summary of results	MPPG 5.a tolerance
8	Output for reference conditions and various standard cutout sizes	Output for reference conditions agrees within 0.5% Some small cutout output factors differ from measurements by > 5%	None listed
8.1a	Small custom cutout	Output error > 5% for 6MeV, 105 cm SSD. All other output factors agree within 3% Crossline profiles too wide^a^	3%/3mm
8.1b	Large custom cutout	All output factors agree within 2%	3%/3mm
		All profile and PDD comparisons resulted in gamma passing rates > 95%	
8.2	Oblique incidence	Absolute dose measurements agree within 2% All profile and PDD comparisons resulted in gamma passing rates > 95%	5%
8.3	Heterogeneous media (cork)	Absolute dose measurements agree within 5% Distance to agreement near R_50_ was < 3 mm	7%

a The calculated crossline profiles for the small custom cutout were wider than measured for one of our TrueBeam machines. However, when this validation test was repeated on a matched TrueBeam linear accelerator, the profile agreement was excellent with passing rates > 95%. Measurement error or a small mismatch between the size of the cutout in the planning system vs. the physical cutout is suspected.

### 3.2 Complex phantom validation

**Figure 3:**
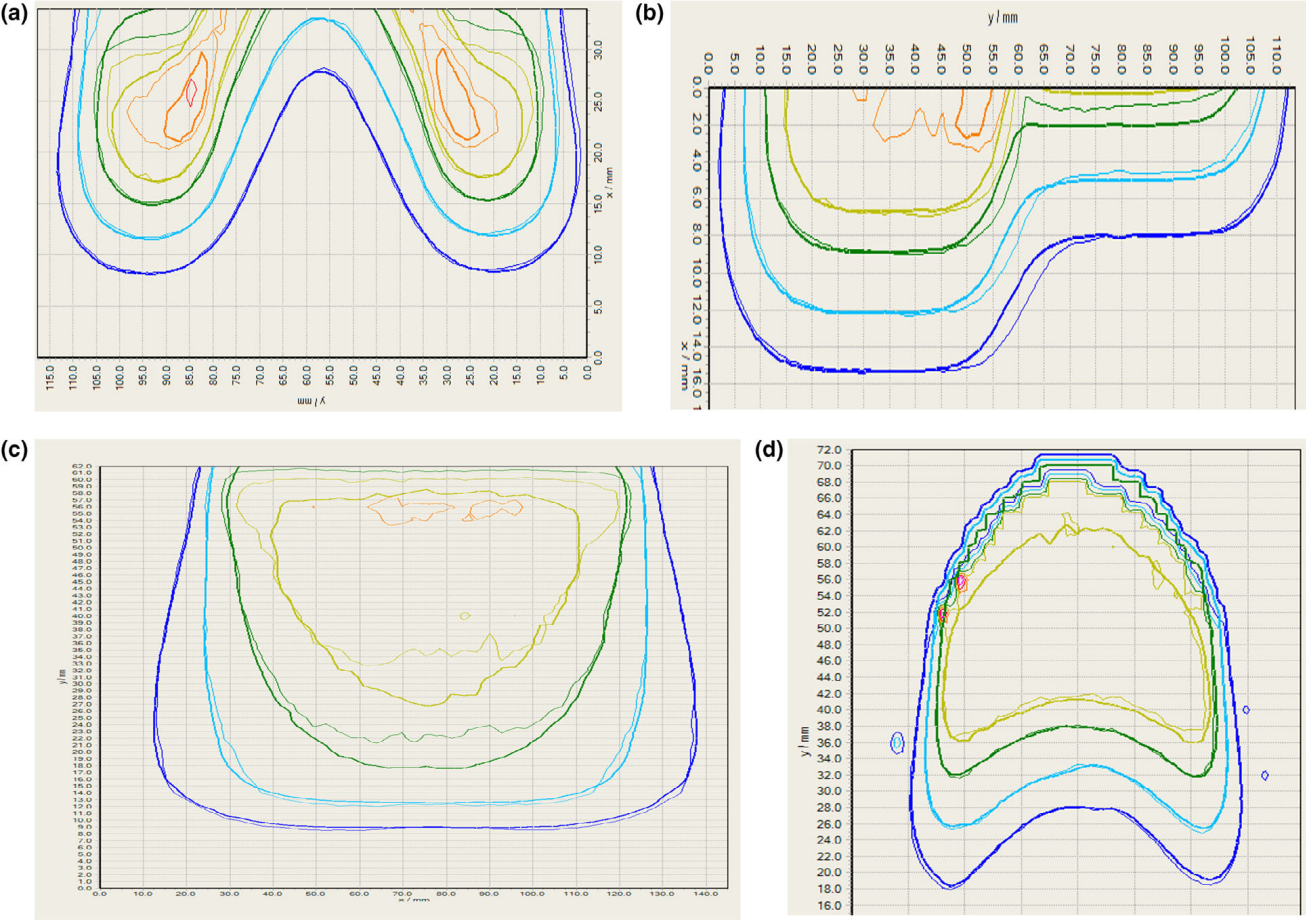
Comparison between RayStation‐calculated dose (thick line) and film‐measured dose (thin line) for (a) 6 MeV electrons for the nose phantom, (b) 6 MeV electrons for the bone phantom, (c) 6 MeV electrons for the lung phantom, and (d) 9 MeV electrons for the breast phantom.

Table 2 shows the results of gamma analysis comparisons for all electron energies for the four complex validation phantoms. For the energy with the worst agreement for a given phantom Fig. 3 shows the overlay between the film‐measured IDLs and the RayStation‐calculated IDLs. Based on these results, the RayStation electron Monte Carlo algorithm is able to accurately calculate the hot spots due to the triangular nose geometry [Fig. 3(a)], changes in range due to bone [Fig. 3(b)], the effect of a curved patient surface [Fig. 3(d)], and penumbra broadening in lung [Fig. 3(c)]. The average agreement for gamma analysis across all phantoms and electron energies was 89.3% pixels passing for 3%/3 mm criteria.

**Table 2:** Percentage of pixels passing gamma analysis (3%/3 mm global criteria, 10% dose threshold) for comparisons between RayStation dose calculations and film measurements for the four complex validation phantoms.

Phantom	Energy			
	6 MeV	9 MeV	12 MeV	15 MeV
Nose	86.3	94.4	94.5	94.1
Bone	82.5	88.9	93.9	90.8
Lung	85.3	91.6	89.1	87.6
Breast	93.5	78.8	89.5	87.8

The worst agreement values for a given phantom geometry are highlighted in yellow.

### 3.3 Clinical implementation considerations

#### 3.3.1 Statistical uncertainty

Based on our initial experiences with electron planning in RayStation, we chose to perform clinical dose calculations using a 2 mm uniform dose grid and a final dose calculation using 500,000 histories per cm^2^. RayStation reports relative statistical uncertainty for each beam based on the mean uncertainty across all voxels with dose >50% of the max beam dose. The resulting relative statistical uncertainty for these dose calculation settings is <1.0%.

#### 3.3.2 Prescription methods

**Figure 4:**
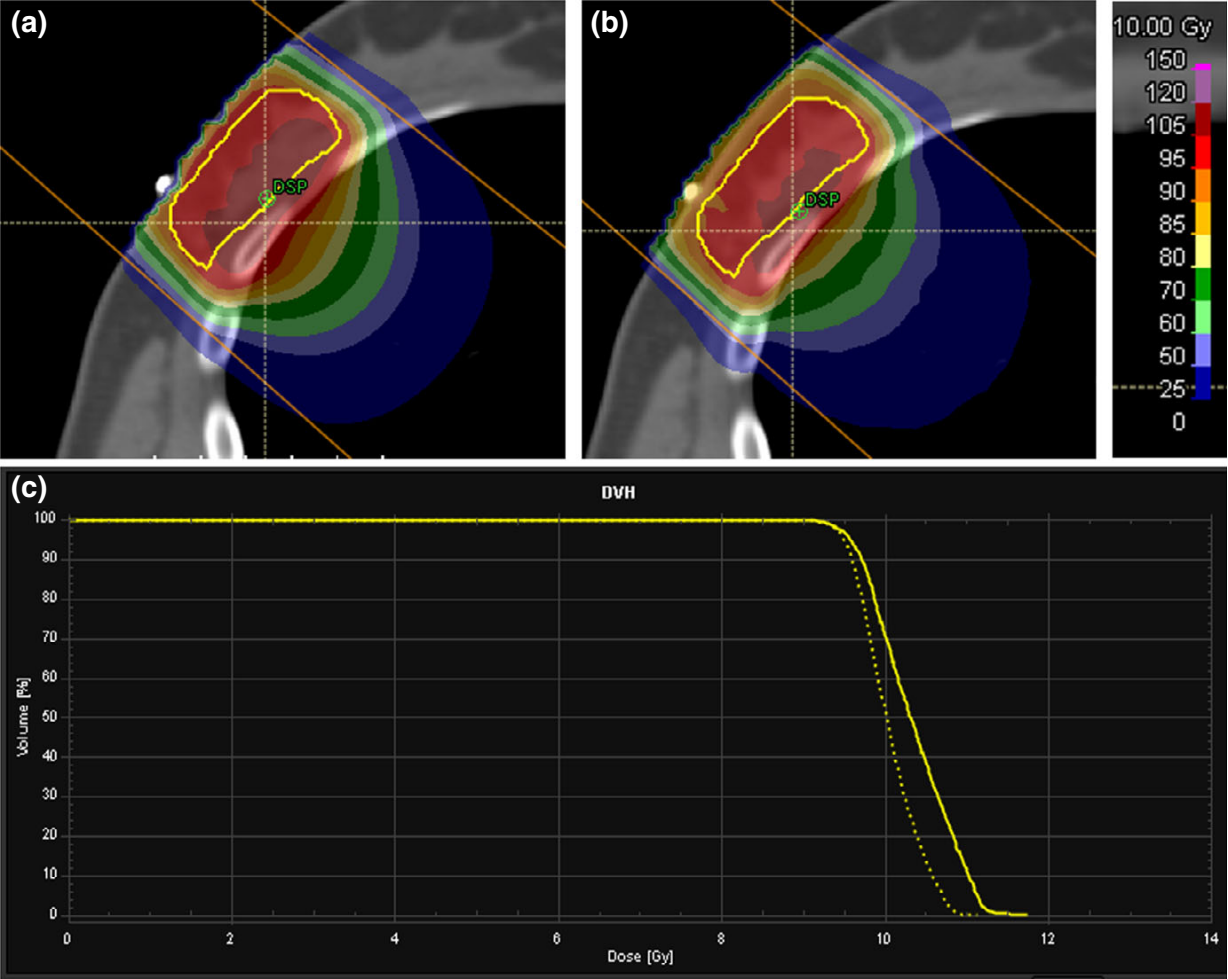
(a) Pinnacle‐calculated dose distribution based on a point‐based prescription for a breast boost treatment plan (the 5th case in Tables 3 and 4), (b) RayStation‐calculated dose based on a volume‐based prescription, and (c) the DVH for the lumpectomy PTV (shown in yellow) for Pinnacle (solid line) and RayStation (dotted line)

Our procedures for electron treatment planning in the Pinnacle planning system utilize a calculation point placed at the depth of dose maximum in order to prescribe dose based on an IDL (e.g., the 90% IDL). However, due to the statistical nature of Monte Carlo dose calculations, prescriptions based on point doses are not recommended by AAPM’s Task Group 105 on Monte Carlo treatment planning. To investigate this issue, several Pinnacle clinical cases were re‐planned in RayStation and point‐based prescription methods from Pinnacle were compared to volume‐based prescription methods in RayStation. Table 3 illustrates examples of volume‐based prescriptions in RayStation that achieved similar coverage as the Pinnacle plans. There were several plans for which there is no contoured target and thus the prescription in the RayStation plan was point‐based. Attempts were made to prescribe to the near minimum dose (D_98_) of an isodose volume (i.e., a contour created based on voxels receiving ≥90% of the maximum dose). However, based on these limited cases, use of a point‐based prescription resulted in MU more similar to the Pinnacle plans. Table 3 also lists the ratio of RayStation MU to Pinnacle MU for these clinical cases; based on this limited dataset there does not appear to be a systematic trend and whether RayStation or Pinnacle MU are higher is likely dependent on both the patient geometry and the prescription method. Based on this initial experience, we recommend that electron plans be created using volume‐based prescriptions whenever there is a contoured target. Figure 4 shows the Pinnacle pencil beam algorithm dose distribution side‐by‐side with the RayStation‐calculated dose for one of these clinical cases (the 5th case shown in Tables 3 and 4), along with a DVH comparison for the PTV.

**Table 3:** Examples of volume‐based and point‐based prescriptions in RayStation vs. point‐based prescriptions in Pinnacle for several clinical cases. IDL = isodose line.

Plan	Energy (MeV)	Cutout dimensions (cm)	RS Rx method	Pinnacle Rx method	Pinnacle MU	RayStation MU	RayStation MU/pinnacle MU
Breast boost	15	8.8 × 8.8	95% of PTV receives 95%Rx	96% IDL	234	236	1.01
Breast boost	12	7.0 × 7.1	95% of PTV receives 95%Rx	96% IDL	242	245	1.01
Breast boost	12	6.9 × 6.6	95% of PTV receives 100% Rx	90% IDL	314	317	1.01
Breast boost	15	8.2 × 5.9	95% of PTV receives 95%Rx	93% IDL	242	255	1.05
Breast boost	9	5.6 × 6.0	95% of PTV receives 95%Rx	90% IDL	257	256	1.00
Chestwall scar boost	6	23.8 × 3.8	90% IDL	90% IDL	244	245	1.00
Forehead	9	6.9 × 6.1	90% IDL	90% IDL	387	403	1.04
Hand	9	15.1 × 14.5	85% IDL	85% IDL	380	365	0.96
Hand	9	7.5 × 9.5	85% IDL	85% IDL	348	338	0.97
Ear	12	6.1 × 4.8	95% PTV receives 95% Rx	90% IDL	398	393	0.99

#### 3.3.3 Density overrides

The RayStation planning system does not take into account the density of structures outside of the external contour in the dose calculation unless the structure is designated as a special ROI type (Fixation, Support, or Bolus). The case shown in Fig. 4 contains a wire over the lumpectomy scar that was present at the time of the simulation scan but will not be present during treatment. Therefore the wire was contoured and overwritten to a density of air for both the Pinnacle and RayStation plans. For cases without bolus, our clinical practice allows the wire structure to either overlap with the external contour and be overwritten to air density (as was done for this particular treatment plan), or to be edited out of the external contour, both options that result in the density of the wire being set to zero. However, for cases with bolus, our clinical practice is to edit the wire out of the external contour, which removes the need to perform a density override of the wire and also makes the bolus created in the planning system conform to the patient contour better (i.e., less puckering). It should be noted that though the wire was accurately contoured and its ROI was overwritten to air density in Fig. 4(b), there is a dip in the 95% IDL for the RayStation dose calculation. This dip in coverage is due to a slight divot in the patient external surface caused by the wire density override based on the assigned densities. This case highlights how sensitive the Monte Carlo algorithm is to even small and localized irregularities in the external surface and how important it is to contour high density objects as accurately as possible when performing density overrides, especially when prescribing to a volume containing local dips in coverage or a calculation point placed in a region with local hot or cold spots.

#### 3.3.4 Secondary MU calculations

Because of the transition to volume‐based prescriptions, our procedures for performing secondary MU calculations, which were previously based on an IDL, also needed to be changed. Several comparisons between RayStation MU and Mobius3D MU were performed for clinical cases. To perform this comparison, a calculation point was placed at the depth of dose maximum in the RayStation plan in order to obtain a %IDL for the secondary MU calculation. Table 4 shows that several of these cases showed greater than 5% differences in MU calculated with Mobius3D vs. RayStation. These differences arise from the different calculation geometries in RayStation, which is able to handle irregular surface contours, heterogeneities, and oblique incidence, and Mobius3D, which performs a calculation in a homogeneous water phantom with normal beam incidence. In order to isolate differences due to electron output for a patient‐specific cutout, a quality assurance plan was created for each patient plan in RayStation. For this quality assurance (QA) plan, the patient‐specific cutout, beam energy, collimator angle, and SSD were used for a dose calculation using a water phantom and a normally incident electron beam. The dose calculated from this QA plan was then used to perform the secondary MU comparison. The use of this water phantom QA plan greatly improved the MU agreement, as expected and summarized in Table 4. The MU then agreed within 2% for all cases. The use of this QA water phantom plan to perform secondary MU calculations was incorporated into our clinical planning procedures in order to have the secondary MU calculation highlight issues with the output calculated in RayStation rather than highlight differences in the calculation algorithms in RayStation vs. Mobius3D. The QA water phantom plan can be easily created in the QA Preparation module in RayStation.

4

**Table 4:** Percent difference between RayStation‐calculated MU and Mobius3D‐calculated MU for various clinical cases. The % difference is shown for the clinical patient plan as well as the QA plan created using a water phantom and a normally incident beam in RayStation. All treatment plans were for 105 cm SSD.

Treatment Plan	Energy (MeV)	Cutout dimensions (cm × cm)	% difference between Mobius MU and RayStation MU	
			Clinical plan	QA water phantom plan
Breast boost	15	8.8 × 8.8	−2.7%	0.7%
Breast boost	12	7.0 × 7.1	−5.4%	0.0%
Breast boost	12	6.9 × 6.6	−2.5%	0.4%
Breast boost	15	8.2 × 5.9	−5.6%	0.0%
Breast boost	9	5.6 × 6.0	−4.2%	0.3%
Chestwall scar boost	6	23.8 × 3.8	1.6%	1.7%
Forehead	9	6.9 × 6.1	−7.2%	0.4%
Hand	9	15.1 × 14.5	5.7%	0.0%
Hand	9	7.5 × 9.5	3.5%	‐0.4%
Ear	12	6.1 × 4.8	−9.2%	‐1.8%

## 5 DISCUSSION

In order to validate electron beam models in the RayStation TPS, we performed the tests recommended by AAPM’s Medical Physics Practice Guideline 5.a. These validation tests, which include comparisons for small and large custom cutouts, oblique incidence, and heterogeneous media, revealed excellent agreement between RayStation dose calculations and measurements. The results confirmed the accuracy of the RayStation electron MC calculation for these situations, with the exception of some small cutout output factors. The MPPG 5.a testing highlighted that small field output factors calculated in RayStation could result in errors >5%. The output error was especially large for the smallest square cutout tested for the 6 MeV electron beam; the error for a 2 cm x 2 cm cutout in a 6 cm x 6 cm applicator was 9.8% for 105 cm SSD and 17.2% for 110 cm SSD. It should be noted that unlike the Pinnacle TPS, which requires the user to input measured output factors for various cutout sizes and SSDs and applies correction factors to the calculated dose to match the measured output exactly, the RayStation planning system does not apply any correction factors. Therefore, the only way to improve the calculation accuracy for output as a function of field size and SSD is to adjust beam modeling parameters. For the 6 MeV electron beam, the virtual source distance parameter was adjusted to try to improve the output agreement. However, adjustments to this parameter did not appreciably improve the output agreement for small cutout sizes and worsened the dose profile agreement. Therefore, the errors in the calculated output for small cutout sizes is a limitation of the RayStation electron dose calculation algorithm of which users should be aware. To identify patient plans in which the calculated output is inaccurate, our clinical procedures state that the output should be measured for a patient‐specific cutout for which the secondary MU calculation disagrees by greater than 5%.

In addition to the validation testing recommended by AAPM’s MPPG 5.a, several complex phantom geometries were also evaluated. The complex validation phantoms were designed to test the dose calculation algorithm in geometries that would highlight the improved accuracy of the more sophisticated Monte Carlo algorithm in comparison to pencil beam algorithms. Thus, the complex validation phantom dataset includes lung and bone heterogeneities, as well as curved and irregular patient surfaces. Comparisons between RayStation dose calculations and film measurements for these complex validation phantoms revealed generally good agreement, with an average of 89.3% pixels passing (3%/3 mm) gamma analysis across all phantoms and electron energies. IDL overlays confirm that the Monte Carlo algorithm was able to accurately calculate hot spots due to the triangular nose geometry, changes in range due to a cortical bone material, the effect of a curved phantom surface, and penumbra broadening in lung. For the breast phantom geometry, gamma analysis comparing film measurements against dose calculations was also performed using Pinnacle’s pencil beam algorithm, and the agreement was poorer than that for RayStation’s MC algorithm (mean of 81.1% pixels passing in comparison to 87.4% pixels passing), as expected.

Beyond dosimetric validation, commissioning of RayStation for electron treatment planning involved several changes to planning procedures, as our clinic was transitioning from a pencil beam algorithm to a Monte Carlo algorithm. Due to the statistical uncertainty inherent in Monte Carlo‐calculated dose distributions, recommendations were made for the number of histories to use for final dose calculations. Additionally, it was recommended that volume‐based prescriptions (e.g., 95% of the PTV receives the prescription dose) be used whenever possible, as volume‐based prescriptions are less sensitive to noise than point‐based prescriptions. Based on several clinical cases, we found that there were generally larger differences for our secondary MU calculation performed using Mobius3D than we were accustomed to for Pinnacle pencil beam calculations. The larger differences are attributable to the more accurate handling of heterogeneities and irregular surface contours with the Monte Carlo algorithm. Therefore, our secondary MU calculation procedures were changed to include a dose calculation performed using the Monte Carlo algorithm for the patient‐specific cutout and a normally incident electron beam on a water phantom. This QA water phantom calculation allows the secondary MU calculation to identify differences in output rather than highlighting differences in the sophistication of the dose calculation algorithm in the primary TPS vs. the secondary check software.

## 6 CONCLUSION

Dosimetric testing of RayStation electron Monte Carlo beam models for a TrueBeam accelerator revealed that though the accuracy was generally excellent, the RayStation beam models exhibited limited accuracy in calculating the output for small cutout sizes. The accuracy of calculated output factors was investigated for various square cutout sizes, applicator sizes, electron energies, and SSDs. There did not appear to be clear trends in the output agreement (e.g., output accuracy always worsens for increasing SSD), and adjustment of beam model parameters did not appreciably improve the accuracy. Despite the limitations in calculation accuracy for small cutout sizes, the Monte Carlo algorithm did result in accurate dose calculations for several challenging geometries that included irregular surface contours and heterogeneities. Clinical implementation of the electron Monte Carlo dose calculation algorithm involved changes to planning procedures to include volume‐based prescriptions for electron plans. Additionally, a water phantom dose calculation using the patient‐specific cutout was incorporated into our secondary MU calculation procedures in order highlight differences in output rather than differences in the sophistication of the primary calculation vs. the secondary calculation.

## CONFLICTS OF INTEREST

The authors have no conflicts of interest to disclose.
